# Effect of Preemptive Flurbiprofen Axetil and Tramadol on Transurethral Resection of the Prostate under Spinal Anesthesia

**DOI:** 10.1155/2016/3942040

**Published:** 2016-02-09

**Authors:** Jinguo Wang, Hongqin Li, Haichun Ma, Na Wang

**Affiliations:** ^1^Department of Urology, The First Hospital of Jilin University, Changchun 130021, China; ^2^Department of Anesthesiology, The First Hospital of Jilin University, Changchun 130021, China

## Abstract

*Objective*. To investigate the efficacy of preoperative intravenous flurbiprofen axetil and tramadol on spinal anesthesia for transurethral resection of the prostate (TURP).* Methodology*. In this prospective clinical study, we enrolled 60 patients undergoing TURP under spinal anesthesia with small-dose bupivacaine and sufentanil. Patients were randomly divided in two: group flurbiprofen axetil and tramadol (Group FT) intravenously received 1 mg/kg flurbiprofen axetil and 1 mg/kg tramadol 20 min prior to the surgical procedures and group control (Group C) was given normal saline. The characteristics of spinal anesthesia, blood pressure, heart rate, analgesic requirement, visual analogue scale (VAS), and overall satisfaction degree were collected.* Results*. Time to the first analgesic requirement was significantly longer in Group FT. Patients who needed postoperative analgesics were fewer in Group FT. VAS scores were lower in Group FT at postoperative time points of 1, 2, 6, and 12 h. The patients in Group FT were more satisfied than in Group C.* Conclusions*. Preoperative flurbiprofen axetil and tramadol can reduce and delay postoperative pain and then decrease analgesic consumption for TURP under spinal anesthesia without an increase of side effects.

## 1. Introduction

Intrathecal anesthesia is widely used for endoscopic urological surgery because it is easy for us to detect early symptom of transurethral resection syndrome (TURS) [1]. Hyperbaric 4 mg bupivacaine and 5 *μ*g sufentanil can provide an adequate spinal anesthetic condition for transurethral resection of the prostate (TURP) and limit the spread of block level but maybe cannot yield an adequate postoperative analgesia [2, 3]. Previous studies have reported that preoperative administration of flurbiprofen axetil or tramadol can reinforce the effect of general anesthesia [4, 5]. However, there are no reports on whether the combination of preoperative flurbiprofen axetil and tramadol which have different analgesic mechanisms can enhance spinal anesthesia and reduce postoperative pain.

The aim of the current study was to investigate the efficacy of preoperative intravenous flurbiprofen axetil and tramadol on low-dose bupivacaine-sufentanil spinal anesthesia for TURP.

## 2. Materials and Methods

Following approval of the institutional ethical committee, 60 patients scheduled for elective TURP were included in the study. Written informed consents were obtained from all patients. Patients with American Society of Anesthesiologists (ASA) higher than II or mental disorders, allergic to the study medications, using analgesics who have liver or renal insufficiency and contraindications to spinal anesthesia were excluded from the study. By a list of numbers generated by a computer and sealed envelopes, patients were equally randomized into two groups: the flurbiprofen axetil and tramadol group (Group FT) and the control group (Group C).

Vital signs were monitored after the patients arrived at the operating room. Venous access was achieved with a 16-gauge cannula. Prior to intrathecal injection, the patients were given 5 mL/kg 0.9% saline, and then infusion was kept at a minimal speed to avoid TURS. All patients were intrathecally given hyperbaric 4 mg bupivacaine and 5 *μ*g sufentanil. The mixed solution was comprised of 0.8 mL 0.5% bupivacaine (bupivacaine, Shanghai Zhaohui Pharmaceutical Co., Ltd., Shanghai, China), 0.1 mL (5 *μ*g) sufentanil (sufentanil, Yichang Humanwell Pharmaceutical Co, Ltd., Yichang, China), and 0.7 mL 10% glucose. Intrathecal puncture was achieved at L3-4 intervertebral space with a 22 G Quincke needle. After we observed the flow of cerebrospinal fluid, the mixed solution was injected for 10 s with the spinal needle bevel oriented to the head, and then the patients were placed in a supine position with heads up 15°. Five min after spinal injection, 1 mg/kg flurbiprofen axetil (flurbiprofen axetil, Beijing Tide Pharmaceutical Co., Ltd., Beijing, China) and 1 mg/kg tramadol (tramadol, Grunenthal GmbH, Aachen, Germany) were administered intravenously slowly over 5 min in Group FT or 0.9% saline was administered in Group C. The surgical procedures started 15 min later. We assessed sensory block level with an alcohol swab on both sides of the midclavicular lines every 2 min until the level reached the maximum and then every 10 min during surgery. The peak sensory block level was identified as the same block level persisting for four continuous assessments. Data associated with sensory block, motor block, analgesic consumption, and hemodynamic variations were collected. Motor block level was scored using Bromage scale (1: complete motor block; 2: almost complete motor block, able only to move the feet; 3: partial motor block, able to move the knees; 4: detectable weakness of hip flexion, able to raise the leg but unable to keep it raised; 5: no detectable weakness of hip flexion, able to keep the leg raised for 10 s at least; 6: no weakness at all). Visual analogue scale (VAS: 0: no pain; 10: the worst pain the patient had ever experienced) and Ramsay sedation scale (RSS: 1: anxious and agitated; 2: cooperative and tranquil; 3: drowsy but responding to command; 4: asleep but responding to tactile stimulation; and 5: asleep and not responding) scores were evaluated at the time points of 0, 1, 2, 6, 12, and 24 h after the completion of surgery. Time to the first analgesic request (tramadol) and the number of patients who required analgesia in the first postoperative 24 h were recorded. When spinal anesthesia was considered inadequate, 100 *μ*g fentanyl was administered intravenously and recorded. In case the patient still complained of pain, general anesthesia was induced and the patient was excluded from the trial. The overall satisfaction degree (poor, moderate, good, and excellent) was also measured and recorded at the end of the study. Adverse effects were recorded during the study period.

The primary endpoint of this study was time to the first analgesic requirement. We assumed that preoperative administration of flurbiprofen axetil and tramadol would extend time to the first analgesic requirement by 30 min, so 23 patients per group were necessary to find differences (5% two-sided *α* and 10%  *β*). We enrolled 30 patients in each group for possible dropouts.

The data were analyzed with SPSS 17.0 (SPSS Inc., Chicago, USA). The analyses of descriptive statistics were performed using Student's *t*-test between the two groups and using variance analysis with repeated measurements within each group. Intergroups differences in block level and VAS score were analyzed using Mann-Whitney *U* test. Categorical data were analyzed with Fisher's exact test. Statistical significance was defined as *p* < 0.05.

## 3. Results

**Figure 1:**
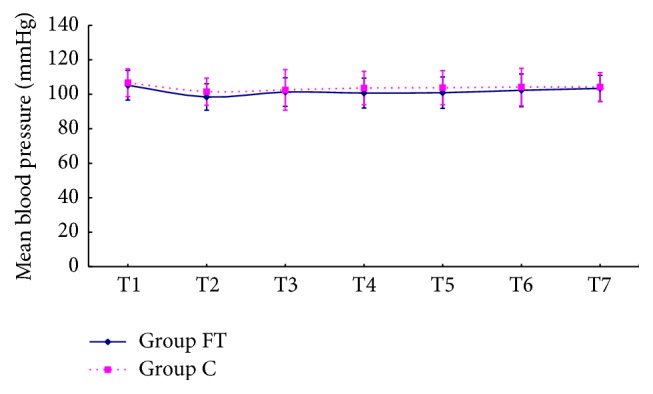
MBP at each time point (T1, basic mean blood pressure, 10 min after arriving at the operating room; T2, 5 min after spinal anesthesia; T3, 10 min after spinal anesthesia; T4, 5 min after drug administration; T5, 10 min after drug administration; T6, 30 min after drug administration; T7, 60 min after drug administration). MBP: mean blood pressure; Group FT: the flurbiprofen axetil and tramadol group; Group C: the control group.

**Figure 2:**
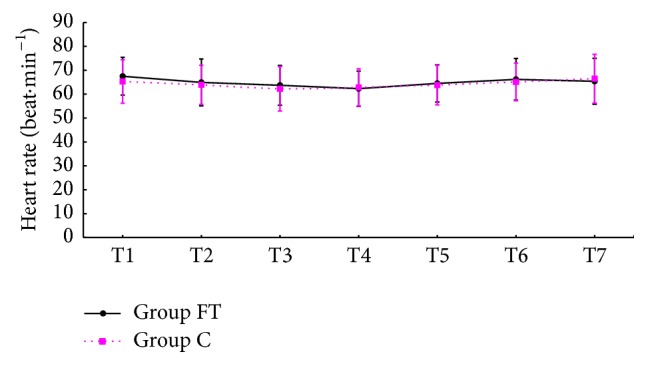
HR at each time point (T1, basic heart rate, 10 min after arriving at the operating room; T2, 5 min after spinal anesthesia; T3, 10 min after spinal anesthesia; T4, 5 min after drug administration; T5, 10 min after drug administration; T6, 30 min after drug administration; T7, 60 min after drug administration). HR: heart rate; Group FT: the flurbiprofen axetil and tramadol group; Group C: the control group.

Fifty-seven patients successfully underwent TURP under spinal anesthesia, and three patients were excluded from this study because of a failure of spinal puncture, one in Group FT, two in Group C.  Table 1 indicated that no significant differences in patients' and surgical characteristics were found between the two groups. Mean blood pressure (MBP) and heart rate (HR) at various time points were showed in Figures 1 and 2. MBP and HR declined because of spinal anesthesia within each group, but not significantly, (*p* > 0.05) and were not different at each time point between the two groups.

**Figure 3:**
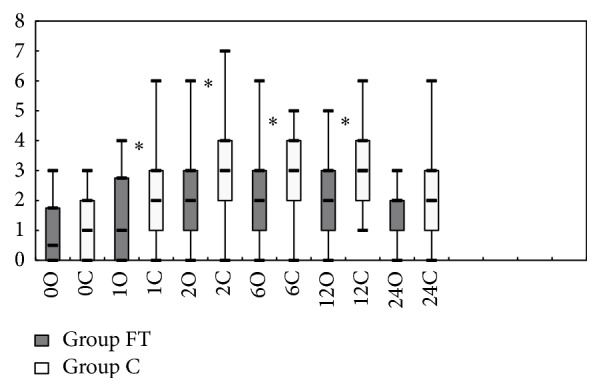
VAS scores at various time points postoperatively. Box plots of postoperative visual analogue scale scores. Results are expressed in median. The top and bottom of each box indicate 75th and 25th percentiles and the error bars minimum and maximum. ^*∗*^ *p* < 0.05 between Group FT and Group C. VAS: visual analogue scale; Group FT: the flurbiprofen axetil and tramadol group; Group C: the control group.

Motor blockade was detected in all patients, but there were no significant differences in the peak level and the duration of motor block between the two groups. Time to two-segment regression was significantly longer in Group FT. Fewer patients required postoperative analgesia and time to the first analgesic requirement was longer in Group FT than in Group C (Table 2). VAS scores were lower at postoperative 1, 2, 6, and 12 h in Group FT (Figure 3).

All patients were observed with sedation score ≤ 3 at each time point. No respiratory depression was found. There was no significant difference with respect to any of the unwanted events between the two groups (Table 3). The overall satisfaction degree was shown in Table 4. The patients in Group FT were more satisfied than the patients in Group C (*p* = 0.028).

## 4. Discussion

Patients for TURP are always elderly with preexisting cardiovascular and respiratory conditions. Thus, it is important to limit the spread of anesthetic block level to decrease cardiopulmonary effects and to shorten recovery time. Spinal anesthesia with small-dose bupivacaine and sufentanil can achieve the goal, but some patients still suffer significant pain after TURP; therefore multimodal analgesia is necessary for this situation [1–3].

Tissue damage resulting from surgical procedures can induce sensitization of the peripheral and central nervous systems and then lead to hyperalgesia and increased postoperative pain [6–8]. Therefore, efforts are directed at inhibiting sensitization of the peripheral and central nervous systems. Alternative strategy involving flurbiprofen axetil with peripheral analgesic function and tramadol having analgesic effect on the central nervous system may be effective for postoperative analgesia in these patients. The result of the current study is consistent with this hypothesis.

Flurbiprofen axetil is not only a nonsteroidal anti-inflammatory drug, but also an injectable nonselective cyclooxygenase inhibitor with peripheral analgesic effect which is also called targeted analgesia. The patented technology of flurbiprofen axetil uses emulsified lipid microspheres that have a high affinity for injured tissues to achieve targeted drug therapy [9].

Tramadol is not only a central effective analgesic with weak opioid features, but also a unique analgesic with double analgesic mechanisms on the central nervous system. Tramadol can alleviate pain by activating *μ* receptor at the cerebral level and magnifying the function of monoaminergic neurotransmitter system, the latter resulting in blocking the transferring of traumatic stimuli at the spinal level [10, 11].

Thus, although neither flurbiprofen axetil nor tramadol has powerful analgesic function, the combination of these two medications can decrease or even erase the peripheral and central sensitivity resulting from the surgical stimuli. The current study indicates that preoperative administration of flurbiprofen axetil and tramadol can prolong time to the first analgesic requirement without an influence on motor blockade. It is possible that preoperative flurbiprofen axetil and tramadol provide these benefits by blunting pain perception, so motor block is not affected. The result is consistent with the previous study [3]. It is a very impressive character, because it will not influence recovery of the patients.

According to this study, MBP and HR do not significantly decrease within each group and are not different at each time point between the two groups. It implies that neither this method of spinal anesthesia nor intravenous flurbiprofen axetil and tramadol is associated with significant hemodynamic variation which is a major concern for elderly patients [2–4].

Major side effects of tramadol are nausea and vomiting. De Witte et al. have reported that the incidence of nausea and vomiting reduced through a 2 min slow infusion administration [10]. It is attributed to 5 min slow injection that the incidences of nausea and vomiting are not higher in the flurbiprofen axetil and tramadol group than the control group. Pruritus reported as a common side effect of intrathecal sufentanil [12] is not a problem in the current study, maybe because elderly patients are not susceptible to pruritus from intrathecal sufentanil [1–3].

One limitation of this clinical trial is the small sample size. Further research with a large number of samples is necessary to evaluate the effect of preoperative flurbiprofen axetil and tramadol on the incidence of chronic pain [13].

## 5. Conclusions

We conclude that preoperative flurbiprofen axetil and tramadol can prolong duration of the first analgesic requirement and reduce postoperative pain without an increase of any side effects.

## Figures and Tables

**Table 1:** Patient demographics and surgical data.

	Group FT (*n* = 29)	Group C (*n* = 28)	*p* value
Age (year)	65.8 ± 8.6	68.6 ± 9.1	0.237
Weight (kg)	64.2 ± 10.2	66.7 ± 11.2	0.379
Height (cm)	167.9 ± 9.8	164.8 ± 8.6	0.247
ASA I/II (*n*)	12/17	15/13	0.784
Duration of operation (min)	55.7 ± 13.5	51.9 ± 12.5	0.275
Prostate volume (g)	58.2 ± 16.7	62.7 ± 13.9	0.274

Data are presented as mean ± standard deviation or number of patients. ASA: American Society of Anesthesiologists; *n*: number of patients; Group FT: the flurbiprofen axetil and tramadol group; Group C: the control group.

**Table 2:** Spinal block characteristics and analgesic consumption.

	Group FT (*n* = 29)	Group C (*n* = 28)	*p* value
Peak sensory block level	T9 (T7–T11)	T9-10 (T7–T12)	0.207
Time to the peak sensory block (min)	12.9 ± 4.3	13.6 ± 4.7	0.559
Time to two-segment regression (min)^*∗*^	115.1 ± 41.5	87.2 ± 38.4	0.011
Peak motor block level	4 (2–6)	3 (1–6)	0.304
Time to peak motor block level (min)	11.3 ± 4.2	12.9 ± 4.6	0.175
Time to Bromage score six level (min)	149.6 ± 39.3	132.9 ± 43.4	0.133
Supplemental fentanyl (*n*)	0 (0.0%)	2 (7.1%)	0.236
Time to the first tramadol request (min)^*∗*^	246.5 ± 51.7	183.8 ± 73.4	0.014
Tramadol analgesia (*n*)^*∗*^	12 (41.4)	21 (75.0)	0.015

Values are presented as number (%), minute, mean ± standard deviation, or median (range). *n*: number of patients; Group FT: the flurbiprofen axetil and tramadol group; Group C: the control group. ^*∗*^ *p* < 0.05 between Group FT and Group C.

**Table 3:** Side effects.

	Hypotension	Bradycardia	Nausea	Dizziness	Pruritus
Group FT (*n* = 29)	5 (17.2%)	4 (13.8%)	6 (20.7%)	3 (10.3%)	3 (10.3%)
Group C (*n* = 28)	3 (10.7%)	2 (7.1%)	4 (14.3%)	1 (3.6%)	2 (7.1%)
*p* value	0.705	0.670	0.729	0.611	1.000

Values are presented as number of patients (%). *n*: number of patients; Group FT: the flurbiprofen axetil and tramadol group; Group C: the control group. ^*∗*^ *p* < 0.05 between Group FT and Group C.

**Table 4:** Patient satisfaction.

	Poor	Moderate	Good	Excellent
Group FT (*n* = 29)	1 (3.4%)	6 (20.7%)	7 (24.1%)	15 (51.7%)
Group C (*n* = 28)	2 (7.1%)	9 (32.1%)	13 (46.4%)	4 (14.3%)

*p *= 0.028, significantly different between the two groups. Values are presented as number of patients (%). *n*: number of patients; Group FT: the flurbiprofen axetil and tramadol group; Group C: the control group.
